# Case Report: Challenges of an extremely delayed diagnosis of classic congenital adrenal hyperplasia in a completely virilized 46,XX patient

**DOI:** 10.3389/fendo.2025.1726368

**Published:** 2025-12-18

**Authors:** Alice Casiraghi, Irene Campi, Silvia Federici, Franco Cernigliaro, Soara Menabò, Luca Persani

**Affiliations:** 1 Department of Medical Biotechnology and Translational Medicine, University of Milan, Milan, Italy; 2 Department of Endocrine and Metabolic Diseases, IRCCS Istituto Auxologico Italiano, Milan, Italy; 3 Department of Radiology, IRCCS Istituto Auxologico Italiano, Milan, Italy; 4 Medical Genetics Unit, IRCCS, Azienda Ospedaliero-Universitaria di Bologna, Bologna, Italy

**Keywords:** congenital adrenal hyperplasia, diagnostic delay, 46,XX disorder of sex development, adrenal myelolipoma, skene gland hyperplasia, case report

## Abstract

Classic Congenital Adrenal Hyperplasia (CAH) due to 21-hydroxylase deficiency is typically diagnosed in early life. We report a 46,XX completely virilized 46,XX patient who was diagnosed with classic CAH at the age of 73 years. He was under follow-up for prostate hyperplasia and referred after the finding of giant bilateral adrenal myelolipomas. He presented with hormonal values initially interpreted as suggestive of hypogonadotropic hypogonadism, prompting further biochemical and genetic analysis. Next-generation sequencing identified heterozygous variants in X-linked genes, uncovering a 46,XX difference of sex development (DSD). Then, *CYP21A2* molecular analysis revealed compound heterozygosity for two pathogenic variants (p.I173N, p.R357W), confirming simple virilizing CAH. The patient’s reticent attitude contributed to the diagnostic delay. However, this unique case reveals the challenges generated by the paraurethral glands hyperplasia - mimicking a prostate due to prolonged untreated hyperandrogenism - as well as the repeated failure to recognize Müllerian remnants on imaging and the critical issues related to diagnostic communication.

## 1 Introduction

Congenital adrenal hyperplasia (CAH) encompasses a group of recessive disorders of adrenal steroidogenesis leading to impaired cortisol biosynthesis. Defective cortisol feedback results in chronic ACTH stimulation of the adrenals, leading to accumulation of upstream precursors and gland hyperplasia (1). These precursors are shunted into the androgen synthesis pathway, leading to hyperandrogenism.

Over 95% of CAH cases result from biallelic loss-of-function mutations in the *CYP21A2* gene encoding 21-hydroxylase, which converts 17-OH-progesterone (17OH-P) and progesterone into 11-deoxycortisol and 11-deoxycorticosterone, respectively. Pathogenic variants in *CYP21A2*, often resulting from gene microconversions or large conversion/rearrangements with its pseudogene *CYP21A1P*, underlie most cases of 21-hydroxylase deficiency, and the residual enzyme activity of each variant largely determines whether patients present with salt-wasting, simple virilizing, or non-classic CAH.

Severe enzymatic defects are classified as classic CAH and include Salt-Wasting CAH (SW-CAH), with cortisol and aldosterone deficiencies causing potentially life-threatening crises, and Simple Virilizing CAH (SV-CAH) with conserved aldosterone activity and variable virilization degrees of 46,XX individuals.

In a female fetus with SV-CAH, excess adrenal androgens during the critical period of sexual differentiation (9–15 weeks gestation) masculinize the external genitalia, causing clitoral enlargement, labial fusion, and rostral migration of the urethral opening. In contrast, internal female genitalia (uterus, fallopian tubes, ovaries) develop normally, as they are Müllerian derivatives and not androgen-responsive.

Nowadays, classic CAH is typically diagnosed through neonatal screening or in early infancy because of adrenal crises in SW-CAH or genital abnormalities in SV-CAH (2), which mainly allow early recognition in females. However, some individuals may still reach adulthood remaining undiagnosed, particularly in regions where neonatal screening has not been implemented.

Here, we report an exceptionally rare case of a 46,XX individual with SV-CAH, whose condition remained unrecognized till 73 years of age. This case is unique due to the prolonged absence of diagnosis despite ongoing medical evaluations for a presumed prostate hyperplasia, the presence of giant adrenal myelolipomas, and the repeated failure to recognize Müllerian remnants on imaging. The patient’s clinical history illustrates the long-term consequences of an untreated disease as well as the critical role played by the unsaid, the misinterpretation of unexpected findings accounting for the dramatic diagnostic delay and the resulting challenges in diagnostic communication.

## 2 Case description

**Figure 1:**
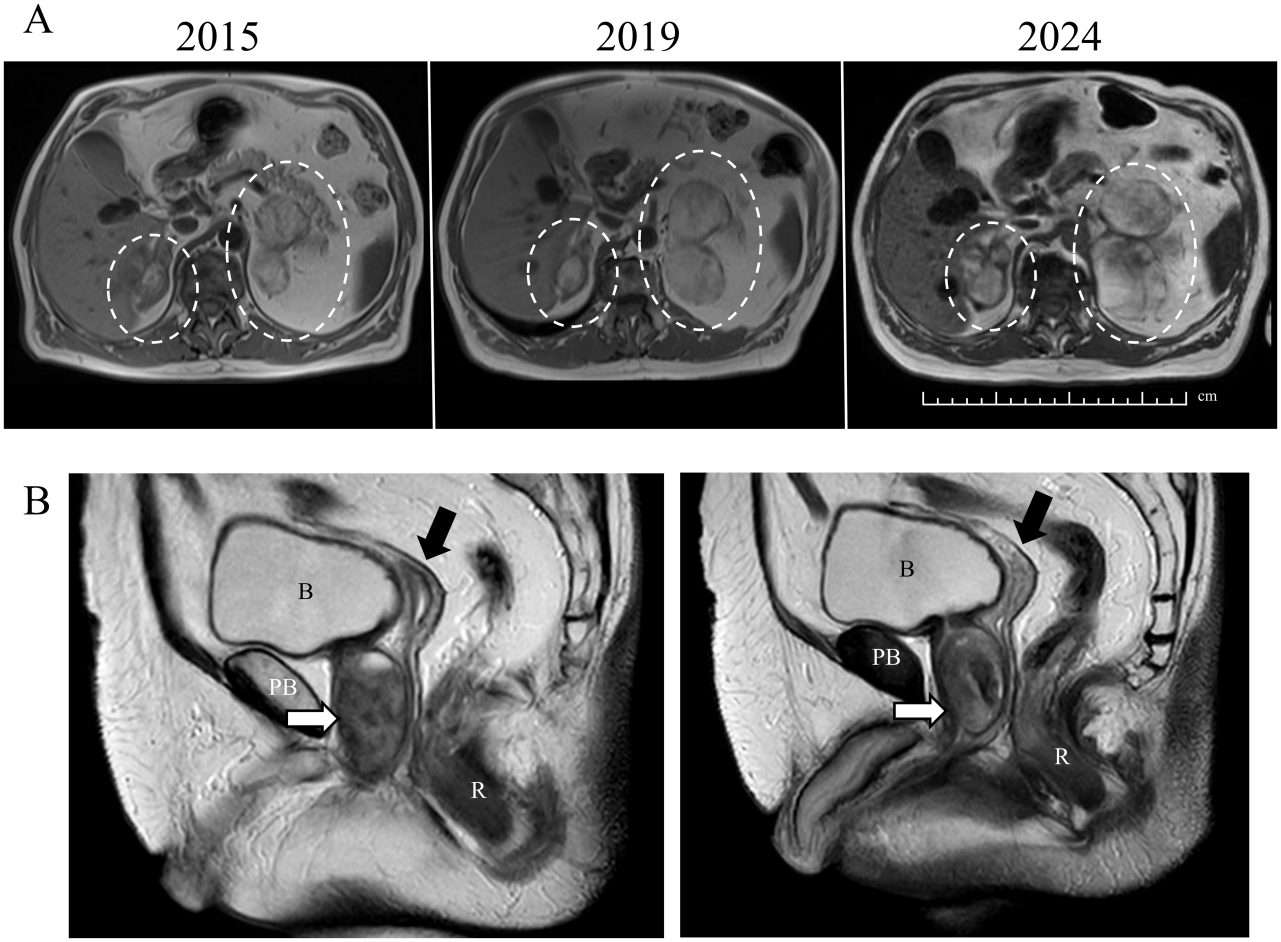
MRI of the abdomen and pelvis. **(A)** shows the progressive increase of the myelolipomas over a 9-year period. The myelolipomas are indicated by dashed white circles. **(B)** displays sections of the pelvis, the prostate-like tissue is indicated by a white arrow and a hypoplastic uterus is marked by a black arrow. B, bladder; PB, pubic bone; R, rectum. These images recall the drawings present in Fibiger report (6).

A urologist referred to our clinic a 73-year-old male with features of longstanding hypogonadism (**Table 1**) and incidental bilateral adrenal lesions found on Magnetic Resonance Imaging (MRI) performed for benign prostate hyperplasia (BPH) and liver cysts (**Figure 1A**) showing liver and prostate. These non-enhanced adrenal nodules measured 30x20 mm (right) and 95x40 mm (left) and were consistent with myelolipomas (**Figure 1A**) showing myelolipomas. Testes were not palpable. He reported explorative surgery for cryptorchidism during childhood, but was unaware of its outcome, and no further actions followed. Although he was the tallest boy in his class at age 10, early growth arrest resulted in a short adult stature (height 150 cm, BMI: 25.2 kg/m2). He never received hormone therapy and denied erectile dysfunction or anosmia. He is married without children but never investigated the causes of infertility. His past medical history was otherwise uneventful except for BPH with abnormal uroflowmetry associated with lower urinary tract symptoms (LUTS) and a PSA of 0.5-0.6 ng/ml (on tamsulosin, 0.4 mg/day). Since age 68, he has experienced tachyarrhythmias without echocardiographic abnormalities, treated with bisoprolol, 2.5 mg/day. The patient gave written informed consent for all subsequent investigations, the sharing of the anonymized results for multidisciplinary consultations and their publication.

**Table 1:** Clinical timeline of the case and in the historical case reported by Fibiger J. in 1905.

Age/Timepoint	Clinical events	
	Present case	Fibiger’ case
Infancy	Surgery for cryptorchidism (no documentation; no known follow-up)	–
10 years	Tallest in class; early growth arrest follows	–
Adolescence	No further height gain; short final adult stature (150 cm)	Short stature (151 cm); not fulfilled his military service
Adulthood		
Gender identity	Male	Male
Social behavior	Behave and socialize as males	Behave and socialize as males. Strong heterosexual attitude. He had several extramarital relationships, according to his wife interview, although he was whispered that he had been not a real man
Marital status	Married to a woman	Married to a woman with many extramarital relationship
Comorbidies	benign prostate hyperplasia, liver cysts, liver steatosis and tachyarrhythmias	Pemphigus from age 37
Referral		
Age (years)	73	47
Reason for referral	Suspect longstanding hypogonadism	Atrophic external genitalia observed at hospital admission due to relapse of pemphigus; died of acute bacterial pneumonia. Autopsy presumably performed to investigate sudden death.
Secondary sex characteristic	Sparse beard Inverted triangular distribution of pubic hair	Scanty full beard of thin fine hair Inverted triangular distribution of pubic hair
External genital apparatus	Small scrotum Absence of vaginal meatus Small penis (<2SD) Hypospadias Unpalpable testes	Small scrotum Absence of vaginal meatus Small penis (4cm) Hypospadias Unpalpable testes
Internal genital apparatus	Patient declined additional investigations; revision of the abdomen MRI by expert radiologist reveals the presence of an atrophic uterus	Normal female pelvic organs
Prostate	At MRI 44x36x39 mm	Well-developed prostate 25x30x10 mm
Adrenal glands	Bilateral adrenal masses at MRI, 30x20 mm (right) and 95x40 mm (left)	Bilateral adrenal enlargement (80x50x30 mm)
Signs of acute adrenal insufficiency	Not reported	Adrenal crisis as a cause of death: unlikely. No macroscopic or histological signs of adrenal crisis. No acute damage to the heart and liver. Red hepatization (right lung)
Total Testosterone	7.4-7.7 nmol/L (nv, males: 9.9-28.0)†	—
LH	0.4 IU/L (nv: 1.7-8.6)†	—
FSH	5.0 IU/L (nv, males: 1.5-12.4)†	
Cortisol	154.5–187.6 nmol/L (nv: 138-690)	—
ACTH	120–204 ng/L (nv: 5-50)	—
17OH-P	88.3 nmol/L (nv, males: 0.6-7.5)†	—
Progesterone	28.9 nmol/L (nv, males: <0.02-0.48)†	—
PSA	0.6 ng/mL (nv in males: 0.1-2.5)†	—
Follow-up		—
	Start of oral glucocorticoid therapy (hydrocortisone)	—
	PSA and testosterone levels decrease; adrenal myelolipomas increase in size (**Figure 1A**)	—

†Post-menopause female reference ranges: LH 7.7 -58.5 U/L; FSH 23.8 -134.8 U/L; total testosterone 0.2-2.4 nmol/L, Progesterone <0.4 nmol/L, 17-OHP 0.4 -1.5 nmol/l

17OHP, 17-Hydroxyprogesterone; ACTH, adrenocorticotropic hormone; LH, luteinizing hormone; FSH, follicle-stimulating hormone; PSA; prostate serum antigen.

## 3 Diagnostic workup

Blood tests were consistent with hypogonadotropic hypogonadism (**Table 1**), with a total testosterone of 7.4 nmol/L (nv, males: 9.9-28.0), LH 0.4 IU/L (nv: 1.7-8.6) and FSH 5.0 IU/L (nv, males: 1.5-12.4) prompting further evaluations. Next-generation sequencing revealed heterozygous single-nucleotide variants in X-linked genes, including a variant of uncertain significance (VUS) p.M1081I in the *IGSF1* gene (Xq26.2) and several heterozygous polymorphisms in the *ANOS1* gene (Xp22.31), consistent with 46,XX difference of sex development (DSD) (3). Indeed, these variants were heterozygous, as expected for a 46,XX individual, whereas a 46,XY individual would be hemizygous for X-linked variants. A CGH array confirmed a female karyotype without any *SRY* gene signal. Further hormonal investigations revealed normal-low cortisol of 154.5 nmol/L (nv: 138-690) with markedly elevated ACTH (120 ng/L nv: 5-50), 17OH-P (88.3 nmol/L nv, males: 0.6-7.5), and progesterone (28.9 nmol/L nv, males: <0.02-0.48), consistent with CAH (**Table 1**). Estradiol levels were normal (99 pmol/L, nv 41-159). Genetic analysis by Sanger sequencing of *CYP21A2* gene revealed two heterozygous pathogenetic variants (p.I173N, p.R357W) associated with classical form, and a VUS in the 3’ untranslated region (c.*13G>A) associated with mild non-classical form. The compound heterozygosity of the two severe variants was previously reported in SV-CAH (4). No relatives were available for segregation study, but likely these variants are in trans configuration, as classic CAH results from biallelic CYP21A2 inactivation. The remaining anterior pituitary function was within normal limits, as was a contrast enhanced pituitary MRI.

## 4 Therapeutic intervention and follow-up

**Figure 2:**
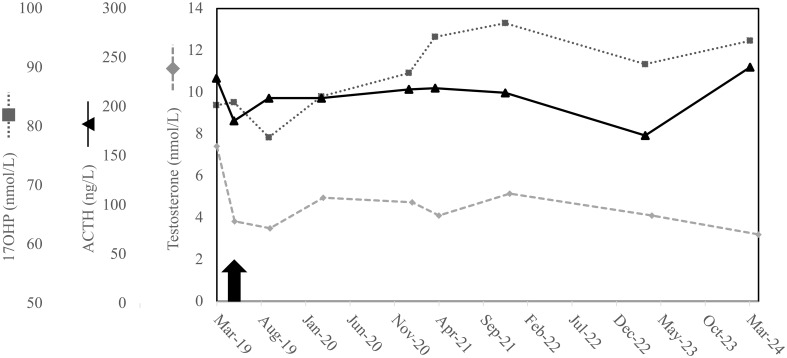
ACTH, cortisol and 17OH-progesterone before and after glucocorticoid treatment. ACTH, 17OH-progesterone (17OHP), and testosterone during the follow-up are represented by solid black line, dotted grey line, and dashed grey line, respectively. The black arrow denotes the starting point of hydrocortisone therapy.

Despite the lack of adrenal crises, prophylactic oral glucocorticoid (OG) replacement therapy was initiated (hydrocortisone 10 mg at breakfast, 5 at noon and 5 at dinner). During treatment, ACTH and 17OH-P did not diminish significantly (after two months of treatment ACTH was 168 ng/L and 17-OHP 85.0 nmol/L), but testosterone decreased to 2.5 nmol/l, (**Figure 2**) and progressive enlargement of adrenal myelolipomas were observed (55 mm - right and 110 mm - left) (**Figure 1A**).

During OG, PSA decreased to 0.2 ng/ml correlating with testosterone. However, this was not associated with any improvement of the LUTS. The prostate likely developed from the paraurethral “Skene” glands due to the prolonged androgen excess, as previously reported in about 16% of untreated 46,XX CAH patients (5). BPH was diagnosed seven years before referral. Uroflowmetry showed a pathological flow curve, with a maximum urinary flow rate of 6 mL per second (normal value: >15 mL/s), an average urinary flow rate of 3 mL per second (normal value: >10 mL/s), a post-void residual volume of 150 mL (normal value: <50 mL), and a voided volume of 122 mL (normal value: >150 mL). At that time, the calculated prostate volume was 20 mL and progressively increased over time. At referral, ultrasound described a prostate of 40x36x37 mm (estimated volume 28 ml) and MRI confirmed a size of 33 ml with intraglandular calcifications (**Figure 1B**). The radiologists unaware of the DSD condition, failed to identify any Müllerian structure on MRI. A review by an expert radiologist informed of the DSD status, revealed a hypoplastic uterus, while atrophic postmenopausal ovaries were not seen (**Figure 1B**). The patient was reluctant to undergo further investigations. Therefore, after consultation with other experts of the European Reference Network for Rare Endocrine Conditions (ENDO-ERN), we decided to inform his NHS general practitioner but not to explicitly disclose the chromosome and gonadal sex status to the patient.

## 5 Discussion

A very similar extreme presentation of CAH was described in an autopsy report in 1905 (6) (**Table 1**). Similarly delayed diagnosis has been reported for other forms of CAH (CYP11B1 defect) in individuals from geographically isolated or rural areas with limited access to healthcare and social isolation (7). In contrast our patient was regularly followed within the healthcare system. Nevertheless, the diagnosis was missed for 73 years, uncovering a still present defect of awareness, even within high-income setting. This case exemplifies how relying on phenotypic presentation when interpreting the clinical and imaging assessment may obscure critical diagnostic opportunities.

This patient underwent surgery for cryptorchidism at an academic hospital, suggesting his care adhered to the standards of the early 1950s, when the term “adrenogenital syndrome” included conditions of hyperandrogenism due to virilizing adrenal tumors or classic CAH, as we know it today (8). At that time, corticosteroid therapy was pioneering, and 46,XX neonates with CAH were often raised as males, since virilization could not be prevented (9). However, any diagnosis possibly shared with the parents was not communicated to the patient, and no caregiver ever suspected a DSD despite the anorchia.

This possible communication gap reflects a generational pattern also observed in a recent survey in women with CAH (10). In this study the oldest participants, who had undergone genital surgery in the 1960s, were least likely to have been informed about their diagnosis or surgical history. At the time, open communication with patients was rare, in contrast to the current emphasis on transparency and shared decision-making promoted by the 2006 DSD Consensus Statement (11). During adolescence, the progesterone and androgens excess, suppressed the hypothalamic–pituitary–gonadal axis and precluded a female pubertal development (12, 13), whereas hyperandrogenism caused peripheral precocious puberty with advanced skeletal maturation and early epiphyseal closure, compromising his final height (1, 14). Later in life, he had no major health issues, however radiological examinations unrelated to gonadal issues were performed at our institution starting in his early 50s and from the age of 60, he regularly attended cardiological visits for primary prevention. Despite these consistent medical contacts and features of longstanding hypogonadism a DSD diagnosis was never considered.

He never underwent pelvic diagnostic exams until the age of 63, when he presented with LUTS. However, no attention was given to anorchia and Müllerian remnants on MRIs were unrecognized at the time, as the hypoplastic uterus was retrospectively identified only 10 years later by our radiologist (**Figure 1B**).

The management of DSDs is often challenging and includes consideration of several individual and socio-cultural factors (15).

One of the challenges relies in the communication of the condition. Communication of DSD diagnosis in adulthood presents specific difficulties, particularly in older individuals. As recently highlighted by Mediå et al., patients diagnosed later in life are more vulnerable to identity disruption, struggling with the integration of new medical information into a long-established self-concept. In contrast, those diagnosed in childhood often demonstrate greater acceptance and reduced psychosocial strain over time (16). In the present case, while maintaining the sex assigned at birth was appropriate, disclosing a 46,XX karyotype at the age of 73 — after a lifetime lived and socially recognized as male — could generate significant psychological consequences. Therefore, we asked the support of other ENDO-ERN experts, and we believe the decision to withhold this information is clinically appropriate and ethically justified, prioritizing the patient’s psychological well-being.

Another challenge relies in the management of the condition. In classic CAH, treatment aims to a) suppress ACTH hypersecretion thus limiting adrenal hyperplasia, reducing excessive androgen action in females and preventing early skeletal maturation in children, and b) prevent adrenal crises (1, 2). The standard therapy consists of relatively high OG doses in 46,XX individuals to diminish androgen excess at the expense of typical adverse effects (1, 2). A novel approach involves ACTH suppression by corticotropin-releasing hormone (CRH) type 1 receptor antagonists, to reduce the OG requirements (17, 18). However, such strategies are not suitable for our patient, who identifies as male, because suppressing androgen production could adversely affect his psychosocial and physical well-being. While acute adrenal crises are rare in SV-CAH, likely because the upstream precursor 21-deoxycortisol can transactivate the glucocorticoid receptor (19), adrenal crises during stressful events remain a considerable risk in classic CAH (2, 20). The presence of giant ACTH-dependent adrenal lesions represents an additional justification for starting OG. Nevertheless, hydrocortisone regimen failed to reduce ACTH and 17OHP, and a slow progression of the adrenal volume was noted. Following surgical consultation, the adrenal masses are under surveillance and surgery will be promptly recommended whenever growth acceleration or compressive signs appear. Under hydrocortisone treatment, the testosterone levels decreased but the patient never reported symptoms of hypogonadism. Dexamethasone or other ACTH-suppressing regimens were considered but raised concerns, also among the ENDO-ERN consultants, regarding the need to add testosterone replacement in a male-identifying patient (1, 2, 15).

An additional challenge is represented by the growth of prostate-like tissue. In this case, testosterone administration could promote the growth of prostate-like tissue (**Figure 1B**), worsening the existing urinary tract obstruction. Although the prostate-like tissue was within the normal male range, LUTS were likely due to its growth in a female-virilized anatomy. A detailed evaluation of the urethra, perineum, and bladder was not performed, so other contributing abnormalities cannot be excluded. The prostate development from paraurethral Skene glands under prolonged androgen excess, is generally overlooked in longstanding females with hyperandrogenism, and no expert consensus exists on the risk of malignancy (1), though two prostate cancer cases were reported in 46,XX CAH patients, including one with bone metastases (21, 22). One of these was on testosterone replacement (21), underscoring the need for biochemical and imaging surveillance in 46,XX individuals chronically exposed to androgen excess, thus including CAH patients and female-to-male gender-affirming hormone therapy (23). Notably, a PSA threshold of 0.1 ng/mL correlates with imaging-detectable prostatic tissue, with good sensitivity and specificity (5).

Interestingly, despite decades of untreated CAH, our patient did not exhibit major metabolic abnormalities. In addition, his bone mineral density was within the reference values for age and male gender, suggesting that long-term hyperandrogenism may have counterbalanced any adverse effect on bone health (1, 2).

In conclusion, the dramatic diagnostic delay in this case illustrates the long-term consequences of untreated 46,XX SV-CAH and reveals several management challenges. This case highlights the potential value of genetic testing in unresolved endocrine conditions and underscores the importance of a sensitive, individualized communication strategy, especially in adult patients diagnosed with DSD.

The prostate-like paraurethral glands’ hyperplasia highlights the risk of misinterpretation when interpreting imaging and may have broader implications for surveillance to these androgen-dependent lesions in all female subjects with prolonged androgen excess.

From the patient’s perspective, the decision not to disclose the 46,XX karyotype was made to protect his psychological well-being, considering a full life lived with a stable male identity. His experience underscores the value of respectful, patient-centered care, even in the context of late diagnosis and complex ethical considerations.

## Data Availability

The original contributions presented in the study are included in the article/supplementary material. Further inquiries can be directed to the corresponding author.
